# Supplementary material for: The MYC axis in advanced prostate cancer is impacted through concurrent targeting of ERβ and AR using a novel ERβ-selective ligand alongside Enzalutamide
Source: bioRxiv. 2023 Nov 17:2023.11.15.567282. Preprint. [Version 1] doi: 10.1101/2023.11.15.567282 (PMC10680693; doi:10.1101/2023.11.15.567282)
Supplement: Supplement 1 [file media-1.pdf]

The MYC axis in advanced prostate cancer is impacted through concurrent targeting of ERβ and AR using a novel ERβ-selective ligand alongside Enzalutamide.

Jaimie S. Gray, Sajad A. Wani, Shahid Hussain, Phoebe Huang, Debasis Nayak,

Mark D. Long, Clayton Yates, Steven K. Clinton, Chad Bennet,

Chris Coss, and Moray J. Campbell

Supplementary tables
Supplementary figure legends

Compound	Class	Function	Low concentration	Medium concentration	High concentration
OSU-ERb-12		ERβ-selective ligand			
Epigallocatechin gallate (EGCG)	DNMT inhibitor	non-nucleoside inhibitor	5	10	20
RG108	DNMT inhibitor	non-nucleoside inhibitor	5	10	20
Decitabine	DNMT inhibitor	nucleoside inhibitor	0.1	0.5	1
Fisetin	DNMT inhibitor	nucleoside inhibitor	1	10	20
GSK126	DNMT inhibitor	nucleoside inhibitor	0.5	1	10
Hydralazine Hydrochloride	DNMT inhibitor	nucleoside inhibitor	5	10	20
Zebularine	DNMT inhibitor	nucleoside inhibitor	5	10	20
FK228	HDAC inhibitor	general HDAC inhibitor	0.1	0.5	1
Entinostat	HDAC inhibitor	HDAC 1 and 3 inhibitor	0.1	0.5	1
Vorinostat	HDAC inhibitor	HDAC 1 and 2 inhibitor	0.1	0.5	1
Enzalutamide	NR antagonist	Androgen receptor antagonist	0.5	1	5
MPP Dihydrochloride	NR antagonist	Estrogen receptor alpha antagonist	0.5	1	5
All trans retinoic acid (ATRA)	NR agonist	Retinoic acid receptor (RAR) agonist	0.1	0.5	1
CD437	NR agonist	RAR? agonist	0.1	0.5	1
ST1926	NR agonist	RAR agonist	0.1	0.5	1
Eicosatetraynoic acid (ETYA)	NR agonist	Peroxisome proliferator-activated receptor gamma (PPARγ) agonist	1	5	10
Bezafibrate	NR agonist	PPARα agonist	5	10	20
Vitamin D3	NR agonist	Vitamin D receptor agonist	0.01	0.05	0.1
AS1842856	transcription factor inhibitor	FOXO1 inhibitor	0.1	0.5	1
CSRM617	transcription factor inhibitor	ONECUT2 inhibitor	0.5	1	5
GN25	transcription factor inhibitor	Snail inhibitor	0.1	0.5	1
Melatonin	transcription factor inhibitor	Inhibitor of OCT4	0.1	0.5	1
BIQ	m6A modulator	Hypoxia inducible factor alpha inhibitor	0.1	0.5	1
Citrate	m6A modulator	AlkB homolog 5 inhibitor	10	50	100
Meclofenamic acid	m6A modulator	FTO protein inhibitor	0.5	1	5
Sinefungin	m6A modulator	S-adenosyl methionine analog	0.1	0.5	1
2D08	other	inhibitor of UBC9 (sumoylation)	1	5	10
Andrographolide	other	NFκB inhibitor	1	5	10
Metformin	other	potential inhibitor of epithelial to mesenchymal transition	12500	25000	50000
PFI1	other	BET bromodomain inhibitor BRD2/4	0.1	0.5	1

Supplementary Table 1. Drugs used in the single and combined proliferation studies in a panel of prostate cancer cell lines.

Transcription.Factor	rank	Enrichment (p.val)
SOX4	1	1.93e-15
ZBTB48	2	2.84e-15
FOXA2	3	5.16e-15
MYC	4	4.9e-12
AFF1	5	5.83e-12
FOXA1	6	6.11e-12
MBOAT4	7	6.7e-12
AR	8	1.5e-11
PR	9	2.08e-11
CEBPB	10	3.35e-11
ERCC2	11	6.05e-11
AGO1	12	7.84e-11
HDAC1	13	1.82e-10
LMNB1	14	1.89e-10
XRN2	15	2.76e-10

Supplementary Table 2: Epigenetic landscape *in silico* analysis of DEGs. RNA-Seq data was analyzed with LISA to identify commonly enriched transcription factors.

ChromHMM	nuc	RX	logPval	Threshold
Poised_Enhancer	NF	E2.Enza	299.22	Significant
Poised_Enhancer	mono	OSU-ERb-12	299.22	Significant
Poised_Enhancer	NF	OSU-ERb-12	299.22	Significant
Poised_Enhancer	NF	Enza	299.22	Significant
Polycomb	mono	OSU-ERb-12	251.81	Significant
Polycomb	NF	Enza	231.15	Significant
Poised_Enhancer	NF	OSU-ERb-12.Enza	221.97	Significant
Poised_Enhancer	mono	Enza	98.47	Significant
Polycomb	NF	OSU-ERb-12.Enza	88.18	Significant
Poised_Enhancer	mono	E2.Enza	79.36	Significant
Polycomb	mono	Enza	75.98	Significant
Polycomb	mono	E2.Enza	50.55	Significant
Transcribed	mono	OSU-ERb-12	41.10	Significant
Active_Enhancer	NF	E2.Enza	40.42	Significant
Active_Enhancer	NF	OSU-ERb-12	40.42	Significant
Polycomb	NF	E2.Enza	38.39	Significant
Polycomb	NF	OSU-ERb-12	38.39	Significant
Active_Enhancer	mono	OSU-ERb-12	29.84	Significant
Active_Enhancer	NF	OSU-ERb-12.Enza	27.18	Significant
Transcribed	NF	Enza	19.61	Significant
Active_Enhancer	mono	E2.Enza	13.34	Significant
Transcribed	mono	Enza	12.82	Significant
Transcribed	mono	E2.Enza	6.39	Significant
Active_Enhancer	mono	Enza	5.41	Significant
Active_Enhancer	NF	Enza	4.37	Significant

Supplementary Table 3: Significant enrichment of drug-induced chromatin accessibility cistromes in ChromHMM defined epigenetic states. 22Rv1, cells in triplicate were treated with vehicle, Enza (1 μM) plus OSU-ERb-12 (100 nM), or the individual treatments, ATAC-Seq undertaken and nucleosome spacing determined (nucleosome free (NF), or mono-nucleosome (mono) and differential enriched binding sites for the combined treatments compared to individual treatments (p.adj < .1) were identified by `csaw` and were overlapped using `bedtools` with ChromHMM regions identified in LNCaP, and enrichment tested with a hypergeometric test (lower.tail = FALSE).

ChIP Target	Direction	Nearest Gene	Class	logPV.h
MYC.K27	Gain	ZBTB48	CoA	3.48
MYC.K27	Gain	PHF13, MIB2	Mixed	3.21
MYC.K27	Gain		Mixed	3.21
MYC	Gain	CAMTA1, GABPB2	CoA	1.52
MYC	Gain		CoA	1.52
MYC	Gain	CHD5	Mixed	1.41
AR	Gain	TBX19	CoA	1.33

ChIP Target	Direction	Nearest Gene	Class	logPV.h
AR.K27	Loss	ESRRG	NR	3.56
AR.K27	Loss	RNF2	CoR	1.19
AR.MYC	Loss	PMF1, RBBP5	CoA	1.02
AR.MYC	Loss		CoA	1.02
AR.MYC	Loss	KDM4A, OTUD7B, CIART, SETDB1, MDM4	CoR	1.0
AR.MYC	Loss		CoR	1.0
AR.MYC	Loss		CoR	1.0
AR.MYC	Loss		CoR	1.0
AR.MYC	Loss		CoR	1.0
AR.MYC	Loss	RXRG	NR	1.0

Supplementary Table 4: Enrichment of coregulators and nuclear receptors in AR, MYC and H3K27ac cistromes in response to ERb12 plus Enza. The indicated cistromes following ERb12 plus Enza compared to either treatment alone were identified by `csaw`, and separated into sites that were gained (upper) or lost (lower), the sites were intersected (minimum of 1bp) and the genomic regions in each intersection annotated to genes within 100 kb, and filtered as to whether the genes were either coactivator (CoA), corepressor (CoR), mixed function coregulators (Mixed), Transcription factors (TF) or Nuclear Receptor (NR). Enrichment was then determined by hypergeometric test and the group gene member closest to a region is indicated (more than one indicated is for regions at the TSS).

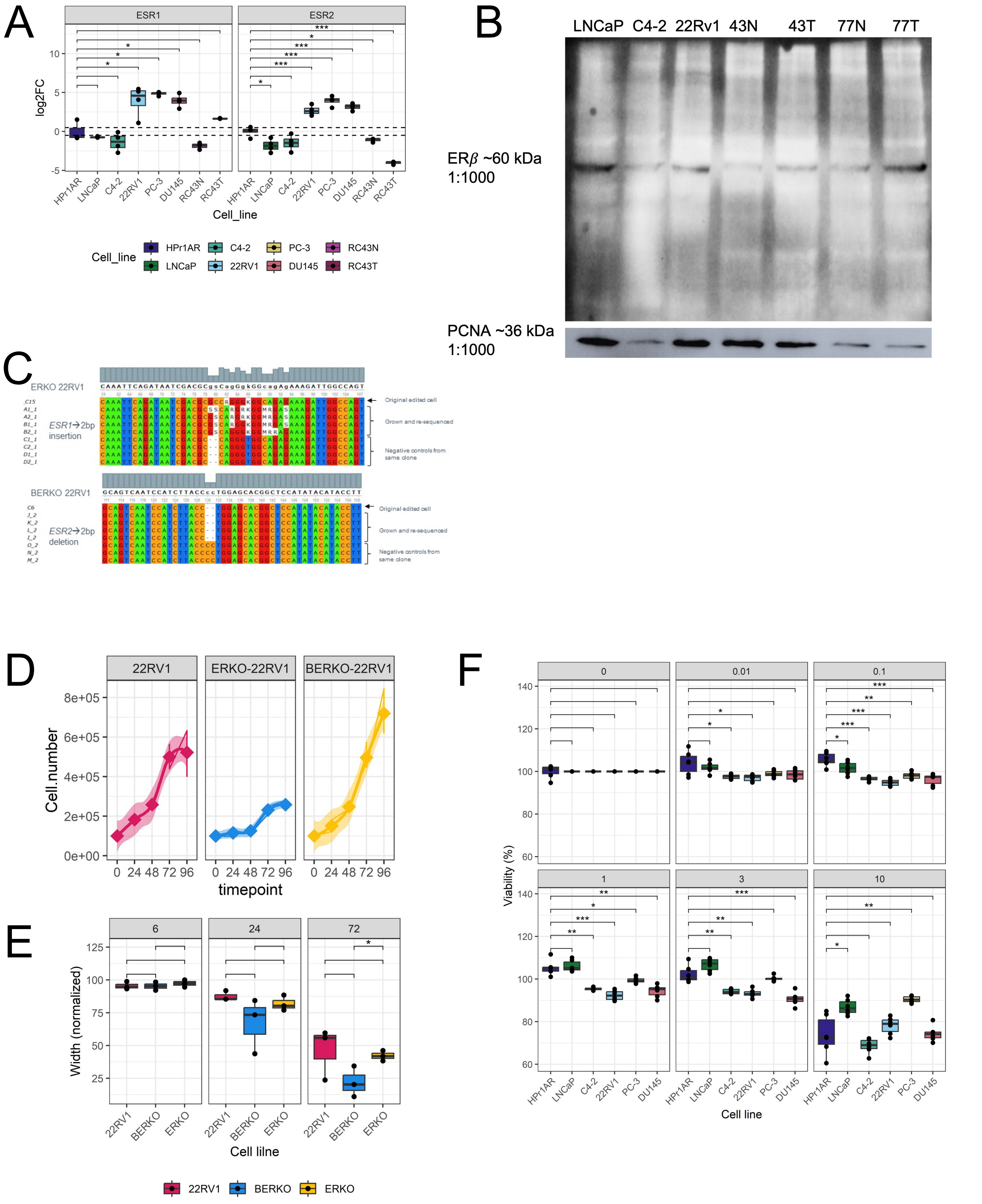

Supplementary Figure 1

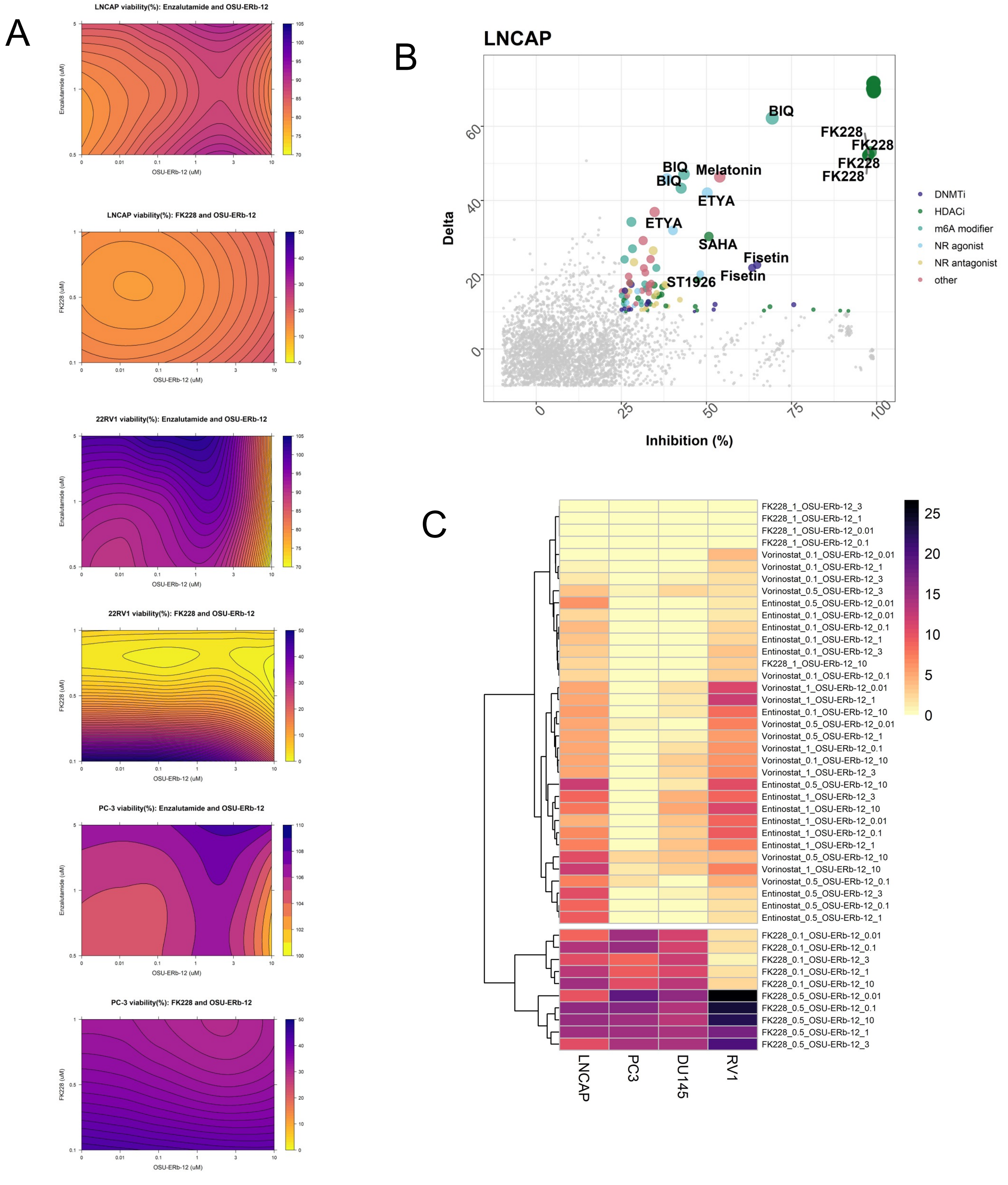

Supplementary Figure 2

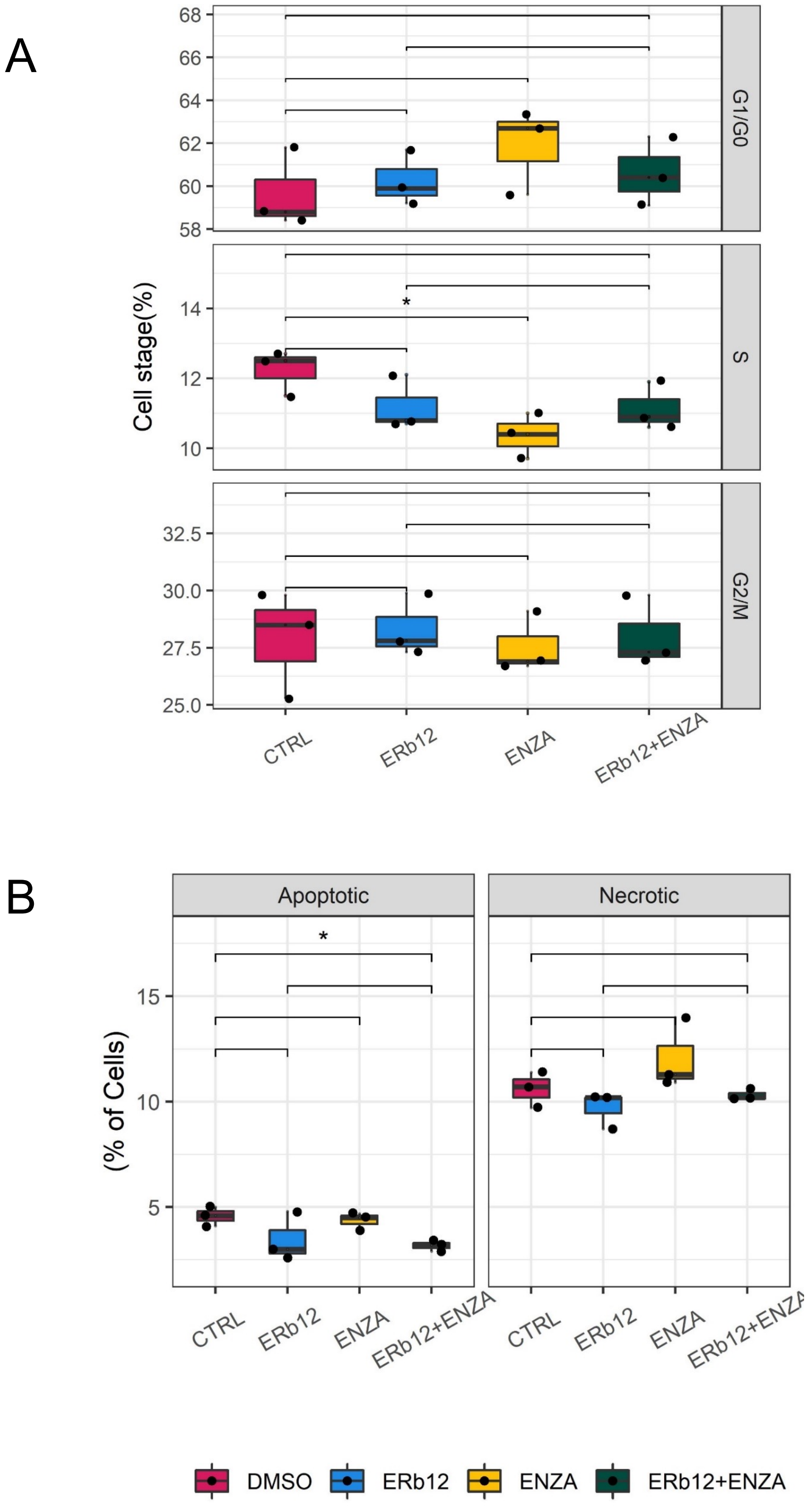

Supplementary Figure 3

A

B

C

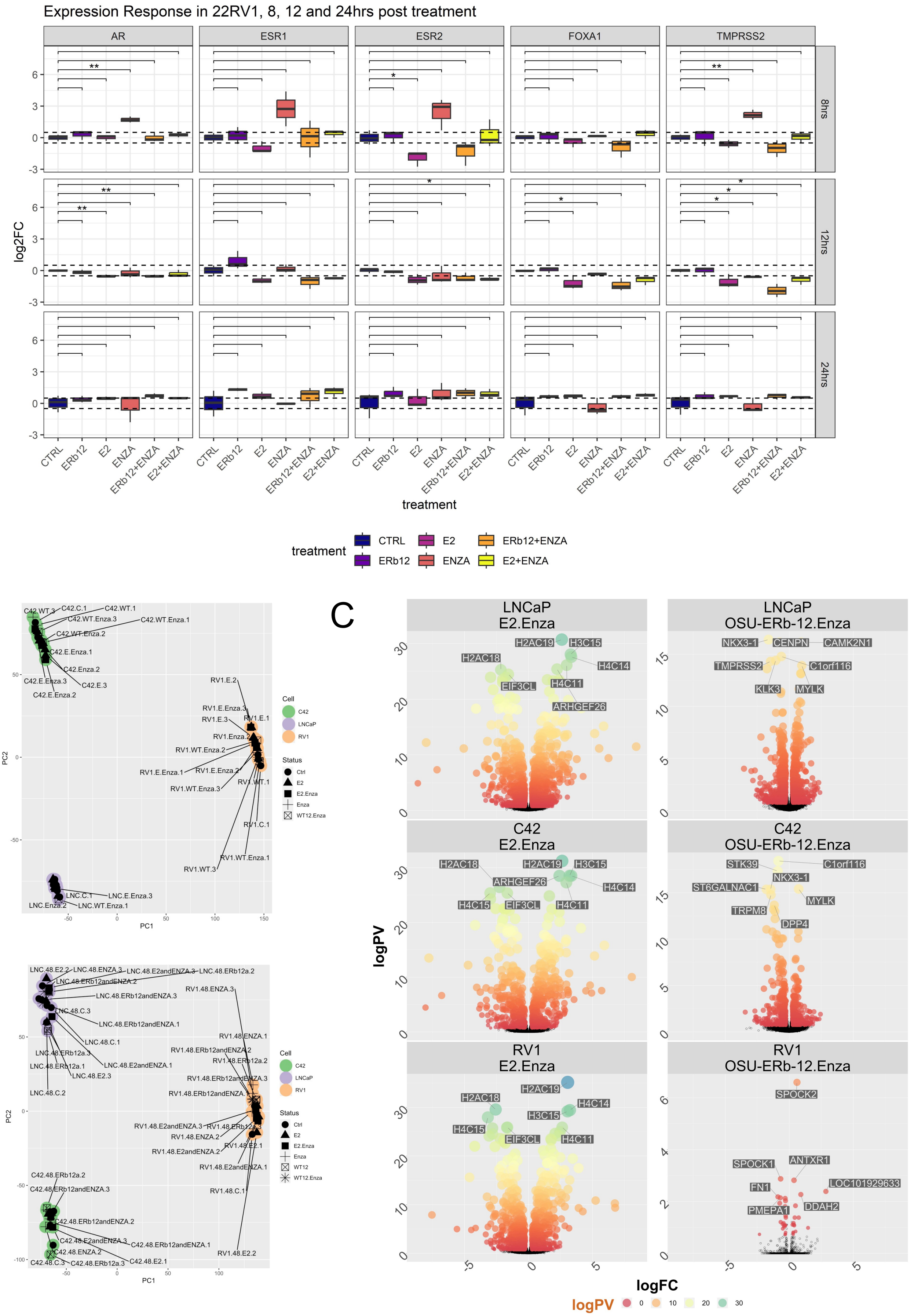

Supplementary Figure 4

A

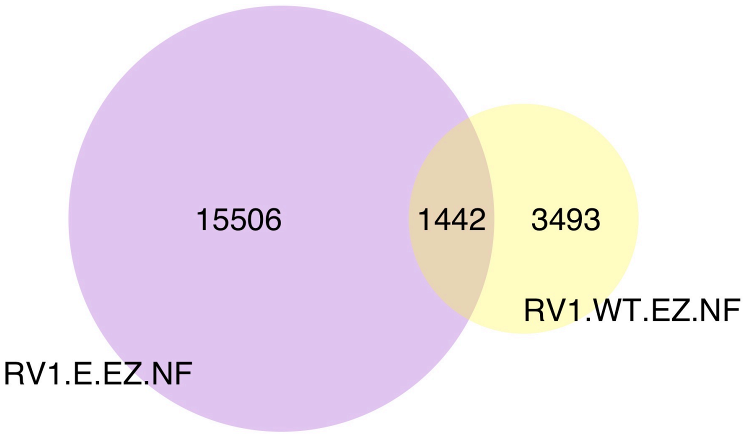

B

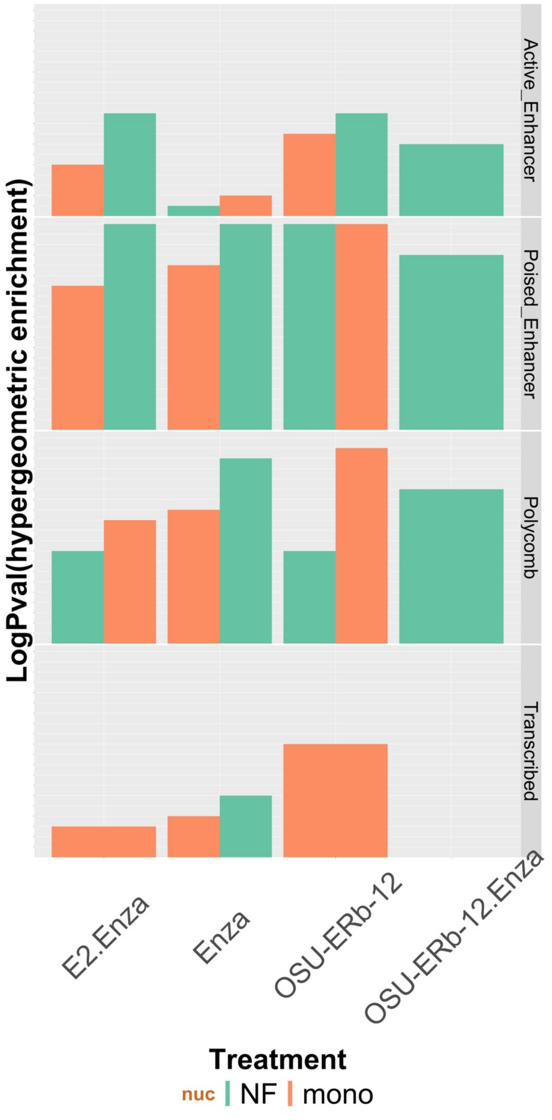

Supplementary Figure 5

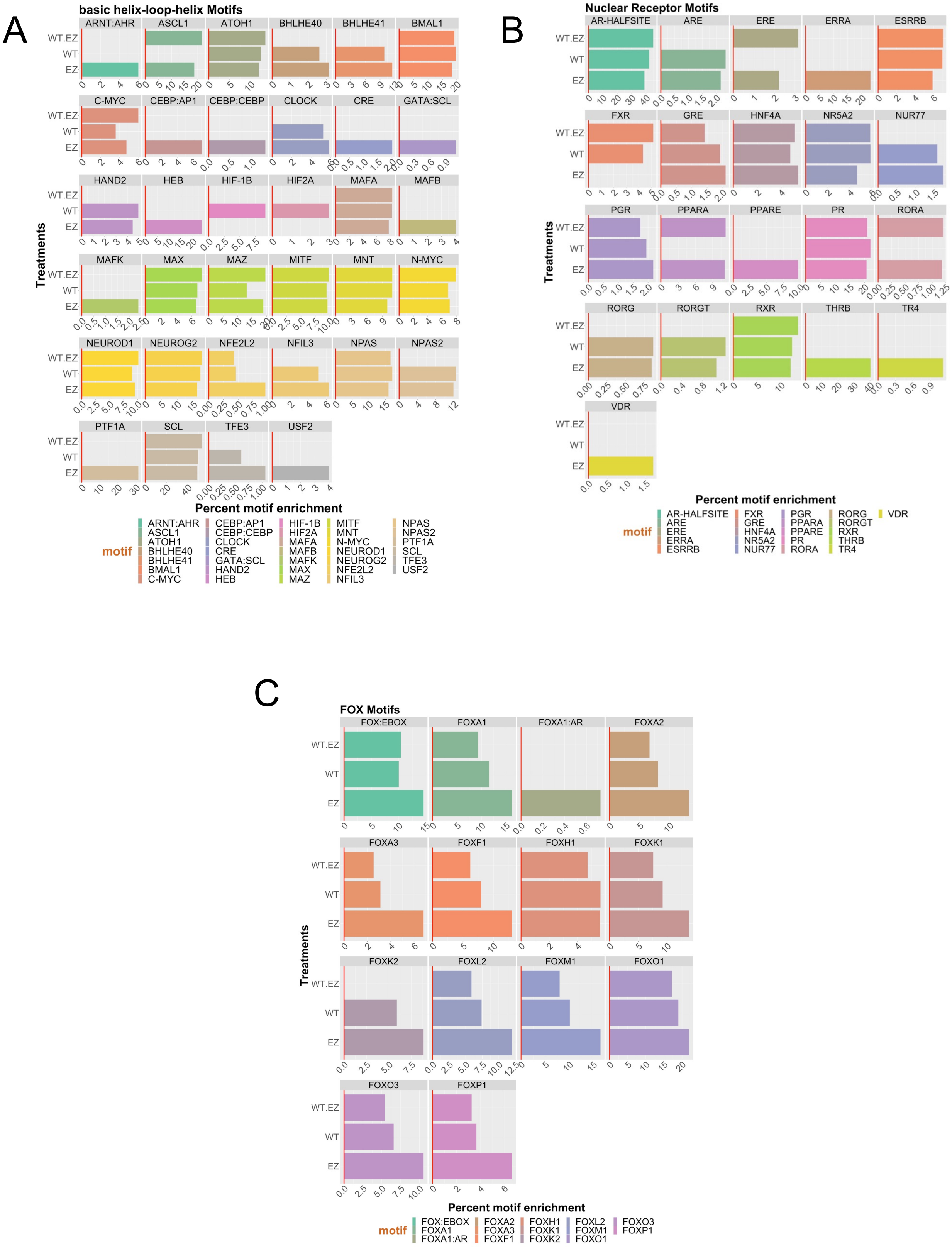

Supplementary Figure 6

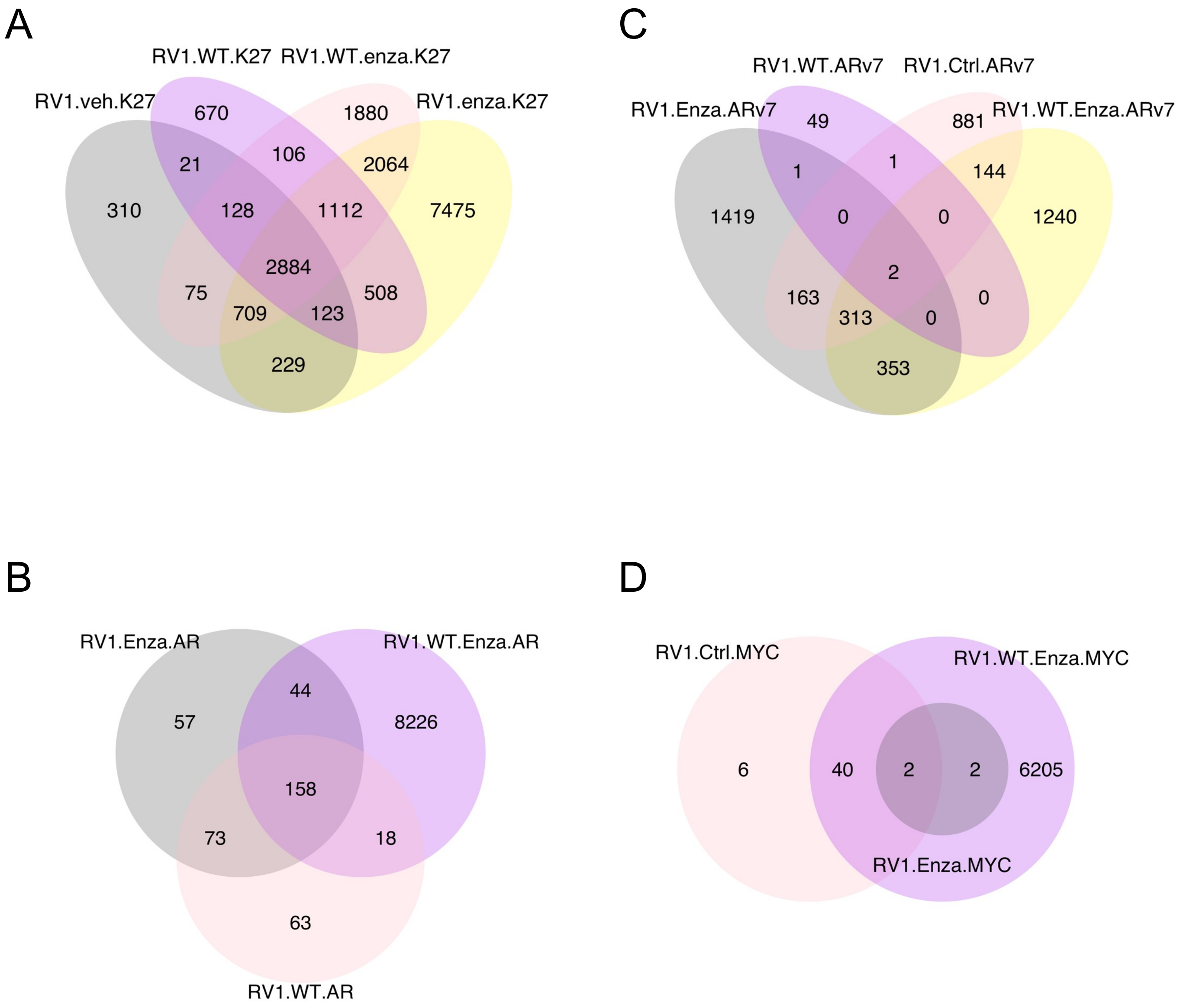

Supplementary Figure 7

A

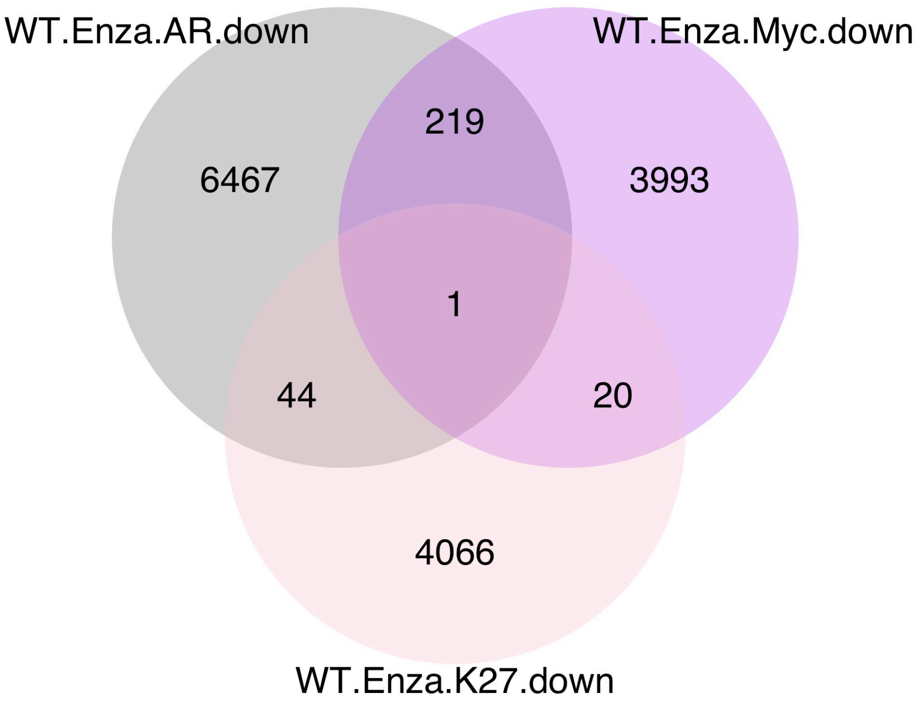

Supplementary Figure 8
